# Feasibility and Effectiveness of an Intervention to Reduce Intimate Partner Violence and Psychological Distress Among Women in Nepal: Protocol for the Domestic Violence Intervention (DeVI) Cluster-Randomized Trial

**DOI:** 10.2196/45917

**Published:** 2023-08-15

**Authors:** Rachana Shrestha, Diksha Sapkota, Devika Mehra, Anna Mia Ekström, Keshab Deuba

**Affiliations:** 1 Public Health and Environment Research Center Lalitpur Nepal; 2 Knowledge to Action Lalitpur Nepal; 3 Griffith Criminology Institute Queensland Australia; 4 Mamta Health Institute for Mother and Child New Delhi India; 5 Medeon Science Park Malmo Sweden; 6 Department of Global Public Health Karolinska Institutet Stockholm Sweden

**Keywords:** intimate partner violence, intervention, violence prevention, mental health, cluster-randomized trial, depression, anxiety, posttraumatic stress disorder, low-income country, women, Nepal

## Abstract

**Background:**

Intimate partner violence (IPV) disproportionately affects people in low-and middle-income countries (LMICs), such as Nepal. Women experiencing IPV are at higher risk of developing depression, anxiety, and posttraumatic stress disorder. The shortage of trained frontline health care providers, coupled with stigma related to IPV and mental health disorders, fuels low service uptake among women experiencing IPV. The Domestic Violence Intervention (DeVI) combines the Problem Management Plus counseling program developed by the World Health Organization with a violence prevention component.

**Objective:**

This study aims to implement and evaluate the feasibility, acceptability, and effectiveness of DeVI in addressing psychological distress and enabling the secondary prevention of violence for women experiencing IPV.

**Methods:**

A parallel cluster-randomized trial will be conducted across 8 districts in Madhesh Province in Nepal, involving 24 health care facilities. The study will include women aged 18-49 years who are either nonpregnant or in their first trimester, have experienced IPV within the past 12 months, have a 12-item General Health Questionnaire (GHQ-12) score of 3 or more (indicating current mental health issues), and have lived with their husbands or in-laws for at least 6 months. A total sample size of 912 was estimated at 80% power and α<.05 statistical significance level to detect a 15% absolute risk reduction in the IPV frequency and a 50% reduction in the GHQ-12 score in the intervention arm. The health care facilities will be randomly assigned to either the intervention or the control arm in a 1:1 ratio. Women visiting the health care facilities in the intervention and control arms will be recruited into the respective arms. In total, 38 participants from each health care facility will be included in the trial to meet the desired sample size. Eligible participants allocated to either arm will be assessed at baseline and follow-up visits after 6, 17, and 52 weeks after baseline.

**Results:**

This study received funding in 2019. As of December 29, 2022, over 50% of eligible women had been recruited from both intervention and control sites. In total, 269 eligible women have been enrolled in the intervention arm and 309 eligible women in the control arm. The trial is currently in the recruitment phase. Data collection is expected to be completed by December 2023, after which data analysis will begin.

**Conclusions:**

If the intervention proves effective, it will provide evidence of how nonspecialist mental health care providers can address the harmful effects of IPV in resource-constrained settings with a high burden of IPV, such as Nepal. The study findings could also contribute evidence for integrating similar services into routine health programs in LMICs to prevent IPV and manage mental health problems among women experiencing IPV.

**Trial Registration:**

ClinicalTrials.gov NCT05426863; https://clinicaltrials.gov/ct2/show/NCT05426863

**International Registered Report Identifier (IRRID):**

DERR1-10.2196/45917

## Introduction

### Background

**Figure 1:**
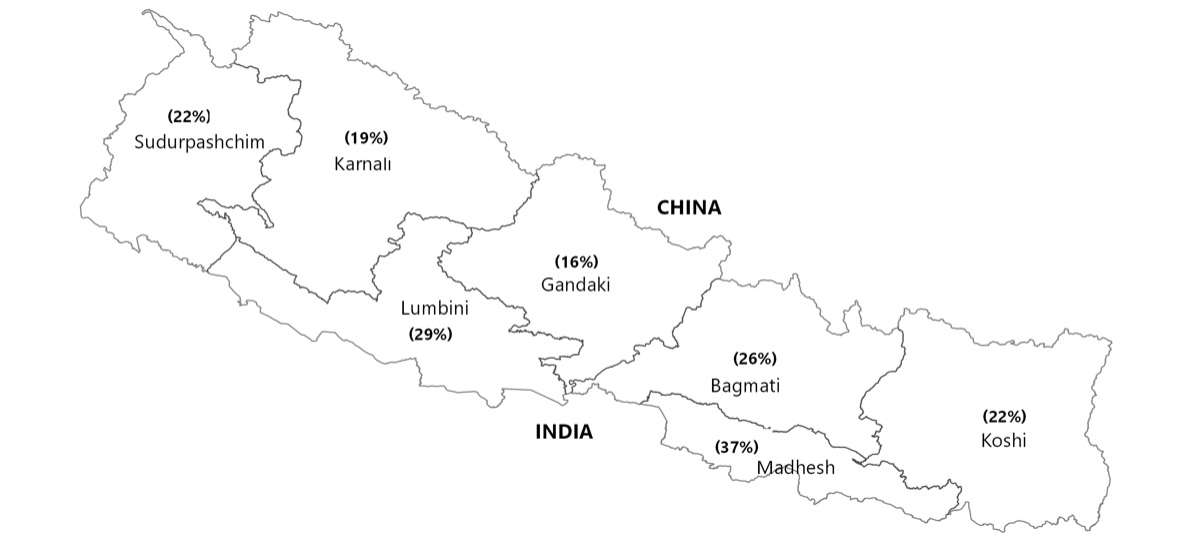
Map of Nepal showing the percentage of married girls and women aged 15-49 years who have experienced physical, sexual, or emotional violence committed by their spouse [6].

Intimate partner violence (IPV) against women is a significant public health problem worldwide [1]. IPV encompasses various behaviors, such as physical, psychological, and sexual harm or controlling behavior, primarily perpetrated by male partners/husbands, ranging from verbal abuse to assaults and even murder [2]. Worldwide, one-third of women experience physical or sexual violence, often perpetrated by their intimate partners [3]. The COVID-19 pandemic has exacerbated this problem, with approximately 50% of women aged 15-49 years reporting that they either experienced violence themselves or knew someone who did [4]. Low- and middle-income countries (LMICs), including Nepal, witness higher rates of IPV. During the first wave of the pandemic, gender-based violence among women and girls increased by 2.7% in Nepal compared to the prepandemic period [5]. A nationwide survey conducted in Nepal found that 26% of ever-married women aged 15-49 years had experienced physical, sexual, or emotional violence from their husbands/partners at least once in their lives, with physical violence being the most prevalent form (23%) of IPV (Figure 1) [6]. Among Nepal’s 7 provinces, Madhesh Province (formerly known as Province 2) has the highest prevalence (37%) of spousal violence/IPV [6].

IPV is a prevalent form of violence against women and is linked to various adverse health and socioeconomic outcomes for survivors. Women who experience IPV are 2.5 times more likely to develop mental and neurological issues, such as depressive symptoms and disorders, suicidal ideation and behavior, and poor psychological adjustment, than those who have not experienced IPV [2]. A systematic review found that pregnant women who have experienced domestic violence (IPV or violence perpetrated by another family member or both) are 3 times more likely to experience postnatal depression [7]. Moreover, there is an increased risk for mental health problems, communicable diseases, health risk behaviors, and reproductive health issues, including pregnancy-related complications [2,7]. A cross-sectional study conducted in Nepal in 2017, involving 397 married women, reported that those who had experienced IPV at any point in their lives were nearly 5 times more likely to have depression compared to those who had never experienced IPV [8]. Common mental disorders significantly contribute to functional impairments and disabilities and severely impact one’s quality of life [9]. Recent studies have demonstrated a bidirectional relationship between mental health issues and victimization. For example, research linking depression and posttraumatic stress disorder (PTSD) resulting from IPV also indicates an increased risk for future victimization [10].

Despite increasing recognition of the mental health and social needs of women who have experienced or are experiencing IPV, the programs or interventions aimed to address their needs are still in infancy, particularly in resource-constrained countries. The literature indicates that the shortage of trained health care providers, leading to poor recognition of violence against women, particularly IPV episodes, coupled with the reluctance of women to disclose and seek support for their experiences with violence has made it difficult to manage IPV and its consequences [11-13]. About two-thirds of the women experiencing violence in Nepal never seek help or tell anyone [6] probably due to low expected support, stigma, and fear that the violence will increase. Mental health is not a national priority in Nepal, reflected by the low availability of mental health services (only 1.5 beds per 100,000 people for in-patient care [14]) and almost nonexistent services in rural areas. Investment in human resources for mental health is extremely low: 0.68 psychiatrists and 0.17 psychiatric nurses per 100,000 people (ie, 200 psychiatrists and 50 psychiatric nurses in total to serve almost 30 million people [14]), which is significantly lower than the average of 6 mental health workers per 100,000 population in LMICs and 70 mental health workers per 100,000 population in high-income countries [15]. This indicates an urgent need for developing interventions that are effective in reducing IPV and associated mental health consequences, while improving access to health care services that do not require highly trained professionals.

On a positive note, growing evidence suggests that psychological interventions delivered by nonspecialist mental health care providers are effective in reducing psychological distress among women experiencing IPV [12]. The World Health Organization (WHO) Mental Health Gap Action Programme includes evidence-based psychological interventions for priority mental health conditions to be delivered by nonspecialists in primary health care settings in LMICs. One promising example developed by WHO is a low-intensity 5-session counseling program known as Problem Management Plus (PM+) [16]. PM+ is a nonintrusive technique for managing stress and solving problems, strengthening social support, and sustaining change. This intervention is based on established cognitive behavioral therapy and problem-solving techniques to manage depression, anxiety, and stress regardless of cause and has been evaluated in randomized controlled trials and found to be effective at reducing psychological distress among women experiencing IPV in Kenya [12] and improving mental health outcomes in conflict settings in Pakistan and earthquake-affected communities in Nepal [17,18].

In high-income countries, several interventions have been introduced to prevent or reduce the effects of IPV [11,19], including counseling interventions, using empathetic listening and psychotherapies, which have demonstrated promising results [11,19,20]. IPV interventions ideally incorporate the development of a safety plan for women to rescue themselves from difficult situations of violence [11,19,20].

The PM+ intervention primarily focuses on managing the consequences of violence on mental health, but it lacks comprehensive components that specifically address the root cause, namely the violence itself. To bridge this gap, the Domestic Violence Intervention (DeVI), proposed in this study protocol, was developed by augmenting PM+ with other violence prevention elements, such as education on the cycle of violence and its impact on health, development of a safety plan, and empowerment-based counseling. Components such as areas of safety, choice making, and problem solving [20,21] were adapted from previous studies [22-24].

The name of the intervention, “DeVI,” an integrated, multicomponent tool, is a neutral term that will be used throughout the trial while communicating with the research team and the participants. In Nepali, and originating from Hinduism, *devi* means *goddess* or *supreme woman deity*.

### Study Aim

This study aims to implement and evaluate the feasibility, acceptability, and effectiveness of DeVI in addressing psychological distress and enabling the secondary prevention of violence for women experiencing IPV through a cluster-randomized trial. The program will be delivered through nonspecialist mental health care providers in public health institutions to increase the feasibility and applicability of such interventions in an LMIC setting. The study will be conducted in Madhesh Province, Nepal, which has the highest-reported prevalence of spousal violence/IPV [6].

### Specific Objectives

The study aims to achieve the following objectives:

- Objective 1: Assess the feasibility of DeVI in health care facilities in Nepal by exploring the views of key stakeholders, health care providers, and local women in the community.
- Objective 2: Train nonspecialist mental health care providers in the delivery of DeVI among women experiencing IPV.
- Objective 3: Explore participants’ and health care providers’ experiences with DeVI and the opportunities and challenges encountered during implementation.
- Objective 4: Measure the effectiveness of DeVI in addressing and resolving psychological distress, reducing the occurrence of IPV, and developing safety strategies for women experiencing IPV.
- Objective 5: Measure the impact of training health care providers in DeVI on their knowledge, attitudes, skills, and stigma toward IPV and the social and health-related consequences of IPV.

### Conceptual Framework of the Study

**Figure 2:**
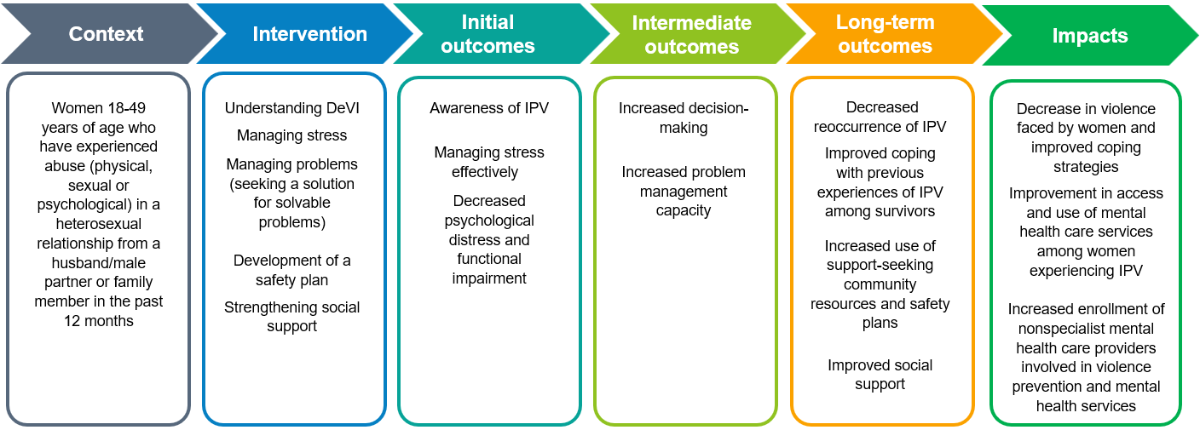
Conceptual framework of DeVI, including expected outcomes. DeVI: Domestic Violence Intervention; IPV: intimate partner violence.

DeVI will be administered to women who have a history of any form of IPV (physical, sexual, or psychological) and are experiencing or have experienced psychological distress. The primary aim of this intervention is to decrease exposure to IPV and IPV-related distress. The intervention aims to enable women to cope with previous experiences of IPV and manage their situation better, strengthen mental well-being, and prevent revictimization (ie, protect participants from future harm). The intervention also aims to aid in the participants’ development of a personal safety plan and strategies, encourage the identification of available support (of any form) in the community, and use it effectively, when needed. Participants who show severe mental impairment, have suicidal thoughts, or are at risk of suicide after participating in the intervention will be handled per the risk management plan. Figure 2 depicts the conceptual framework of this study in brief.

## Methods

### Ethical Considerations

This study was approved by the Ethical Review Board of the Nepal Health Research Council (approval no. 852/2019), and permission was obtained from the Ministry of Health and Population, the Health Directorate of Madhesh Province, and all study sites. The study will follow the *Ethical and Safety Recommendations for Intervention Research on Violence against Women* developed by WHO [25]. Participation in the study is voluntary, and participants have the right to refuse or withdraw at any point without providing a reason. Written informed consent will be obtained from all participants. In cases where participants are unable to read or write, a witness will be present during the consent process. Women needing more time to decide will be asked to communicate their decision during their next health care facility visit. Given the sensitivity of IPV/domestic violence, speaking openly about such experiences can be challenging due to shame, stigma, fear, and pressure. To address this, intervention implementers will receive training to create a comfortable environment where survivors of violence can feel at ease sharing their experiences. Further, the intervention will be conducted in a private room.

### Study Design and Setting

This study is designed as a 2-arm parallel cluster-randomized trial comparing the study outcomes in the intervention arm to those in the usual standard-of-care (control) arm. The study protocol has been devised in accordance with the Consolidated Standards of Reporting Trials (CONSORT) guidelines for cluster-randomized trials [26].

The trial will be conducted across 8 districts of Madhesh Province, Nepal, which has the highest prevalence of IPV and poorer sexual and reproductive health outcomes indicators compared to other provinces [6]. Although the legal age of marriage under Nepali law is 20 years [27], women in Madhesh Province tend to marry 4 years earlier and are less likely to be literate than women in other provinces [6]. Poor socioeconomic conditions, low awareness of personal and human rights, poor quality of life, and traditional norms are contributors to IPV [28] and are common in Madhesh Province.

The study will be carried out in 24 health care facilities (only public hospitals and selected primary health care centers), both rural and urban, since these types of facilities constitute a major proportion of facilities available in the province that provide a range of services to the public and are visited by patients from diverse backgrounds. Some of the selected public hospitals, typically at the district level or higher, also offer integrated services for survivors of violence through 1-stop crisis management centers. These specialized centers offer a platform for women and care providers to exchange information regarding available measures to combat violence against women, as well as referrals for additional legal services, if necessary, and will enhance our comprehension of the effectiveness of interventions within the current context.

A total of 63 frontline female health care providers (eg, nurses and auxiliary nurse midwives [ANMs]) were trained to deliver DeVI: 33 (52%) health workers from the intervention sites and 30 (48%) health workers from the control sites. Of these, 9 (27%) health workers from the intervention sites and 8 (27%) from the control sites received training to be backups for the intervention implementation or data collection if the primary implementor or data collector was unavailable due to unforeseen circumstances, such as transfer to another health care facility, illness, or pregnancy.

### Formative Assessment and Consultation Workshop

A formative assessment was conducted during a preliminary site visit before the commencement of the trial in order to explore the behavior, experiences, opinions, and knowledge of key stakeholders, health care providers, and women experiencing IPV/domestic violence. A qualitative approach using focus group discussions (FGDs), in-depth interviews (IDIs), and key informant interviews (KIIs) was followed to collect data for the formative assessment. The key stakeholders included were women experiencing IPV/domestic violence, local health care providers, women seeking care in the outpatient department, representatives from various community-based organizations, and individuals/experts from federal and provincial levels. A total of 21 interviews, including 15 (71%) IDIs with health care providers, 3 (14%) IDIs with women visiting the outpatient department, and 3 (14%) KIIs with the stakeholders, and 1 FGD with women experiencing IPV were conducted in Nepali by trained field interviewers.

The FGD explored the women’s knowledge, attitudes, and opinions about IPV/domestic violence, as well as personal experiences of violence and its mental, physical, and social impacts. The FGD further explored the challenges these women faced while coping with past experiences of violence, along with barriers to and facilitators of seeking help from a victim’s perspective. IDIs with local health care providers focused on existing models of care for women experiencing IPV/domestic violence and their perceptions, experiences, and challenges in managing IPV/domestic violence. IDIs with women seeking care explored their general perspectives of IPV/domestic violence. KIIs with various key stakeholders from different community-based organizations and local nongovernmental organizations (NGOs) explored their role, perceptions, and challenges in preventing and managing IPV/domestic violence. Furthermore, these interviews explored health care providers’ suggestions and feedback on DeVI’s ability to address psychological distress issues and reduce the recurrence of IPV/domestic violence in communities in which they have been working.

National-level consultation workshops with experts in the field of IPV and mental health were also conducted. Their feedback on the study intervention and tools was acknowledged, and our intervention was updated accordingly. Findings from the formative assessment and consultation workshops were used to adapt the data collection tools and intervention according to the local, social, and cultural context and the participants’ knowledge about the topics. Additionally, these findings helped the study team customize the training to be provided to intervention implementers and data collectors at the local health care facilities.

### Validity and Reliability of the Tools

All the tools used in this study are standardized and have been used and validated previously in South Asian contexts, including Nepal [17, 29-31]. The tools were translated into Nepali and local languages (Bhojpuri and Maithili), which were reviewed by respective subject and language experts. The validity and reliability of these translated tools were further assessed during pretesting. Pretesting was conducted within a small sample of local working teams, including survivors of violence, and the tools were amended accordingly.

### Training of the Study Team

Public health experts, psychiatrists, and staff working on violence against women provided all the field team members (staff nurses and ANMs) involved in the study with specific training in an interactive way, ensuring active participation in the study. The intervention implementers received 10-day training primarily focused on DeVI, the integrated, multicomponent intervention, and were briefed about the types of violence, their prevalence and impact, and the status of women in Madhesh Province. The implementers were also thoroughly trained in implementing the intervention, conducting sessions, and using flipcharts, brochures, worksheets, templates, and forms. In addition, they were trained in the importance and maintenance of confidentiality and ethical considerations. Furthermore, they were trained in how to build trust with the participants, such as approaching them politely, and how to create a comfortable and friendly environment for basic counseling and intervention. While delivering the intervention, the implementers may feel strong emotions and stress, so they were trained to handle their own stress. Although each participant will receive the intervention in a separate, private room at a health care facility, the intervention implementers were also trained to divert the topics to maternal/reproductive health–related issues if interrupted by the person accompanying the participant, such as the woman’s spouse/in-laws. Participants will also be informed about such diversions.

Data collectors for both arms received 3-day training in approaching the participants, following ethical guidelines, maintaining confidentially, determining eligibility, using various tools and techniques for data collection, getting oriented with Research Electronic Data Capture (REDCap) data collection software, and sharing data with the central team. They were also trained in using screening, baseline, and follow-up tools.

### Participants and Recruitment

#### Sample Size

Based on the sample size calculation, a total of 912 eligible women (456, 50%, in each arm) will be included in the study. This sample size for the cluster-randomized trial was calculated considering 80% power to detect 15% absolute risk reduction in the IPV frequency in the intervention arm at a statistical significance level of α<.05 [32]; we estimated a 50% reduction in mental health problems in the PM+ group, as measured with the 12-item General Health Questionnaire (GHQ-12) [33]. The minimum number of clusters required in each arm was 6, which was later increased to 11 to ensure an efficient trial design [34]. The number of clusters was further increased to 12 to account for dropouts at the cluster level [34]. Finally, a total of 24 clusters (12, 50%, intervention and 12, 50%, control clusters) were included, and 38 eligible women from each cluster regardless of the arms will be enrolled in the study.

#### Recruitment

Participants will be recruited using different approaches, including direct recruitment by asking women visiting health care facilities for maternal and reproductive health services by trained ANMs and nurses. In addition, women visiting the hospital-based 1-stop crisis management centers will also be approached for the study. Furthermore, female community health volunteers and community leaders may also disseminate information about the study to the community in any gatherings, such as mothers’ groups, community meetings, or health-related events.

#### Eligibility and Screening

The inclusion criteria for the participants are as follows: (1) women aged 18-49 years; (2) those self-reporting experience of abuse (physical, sexual, or psychological) in a heterosexual relationship, involving their husbands/male partners or other family members, occurring within the past 12 months; (3) women who are nonpregnant or in the first trimester; (4) those with a minimum score of ≥3 on the Psychological Distress Scale (measured with sthe GHQ-12) [35]; and (5) those living with their husbands or in-laws for at least 6 months. Women will be excluded if they (1) have severe cognitive impairment, (2) are seeking treatment for life-threatening emergency care, (3) or are at imminent risk for suicidal ideation and attempt. A questionnaire that helps in determining these eligibility criteria will be formulated and used specifically for the purpose of screening women for eligibility. Women who are at imminent risk for suicidal ideation and attempt will be referred to a psychiatrist.

### Retention of Study Participants

Contact information of the participants, alternative contact persons, and preferred times for making phone calls for safe contact will be collected in order to keep in touch with them. If a participant absconds from a scheduled session or follow-up, the intervention implementer will try to contact her over the phone and encourage her to attend all sessions and follow-ups. If the participant is still absconding or cannot be contacted over the phone, the intervention implementer will contact female community health volunteers to follow up with the participant personally or over the phone. Female community health volunteers properly informed by the intervention implementer will then personally visit the participant at home following ethical guidelines [25]. All ethical guidelines [25] described in detail in the *Ethical Considerations* section will be considered while following up with participants to continue and complete the intervention. However, participants will be clearly informed that they are free to withdraw from the trial at any time without giving reasons.

### Randomization and Blinding

From a sampling frame of 43 health care facilities available in Madhesh Province, a total of 24 (56%) facilities were selected based on the flow of women seeking various health-related services. The selection process involved randomization of health care facilities using a computer-generated random number table, ensuring a 1:1 ratio of intervention and control clusters (12, 50%, intervention and 12, 50%, control clusters). Each health care facility represents 1 cluster.

Participants visiting the intervention sites will be recruited into the intervention arm, while those visiting the control sites will be recruited into the control arm. To maintain blinding, both participants and health care facility staff will be completely unaware of the information pertaining to the 2 different arms, as well as the arm to which their respective health care facility belongs. However, it may not be possible to conceal this information from the study team responsible for the study design. Therefore, the study will be unmasked during data monitoring, evaluation, and analysis.

### DeVI: An Integrated Multicomponent Intervention

This intervention is structured into 5 weekly sessions, where each session lasts for about 90 minutes. The first session includes general information about DeVI, components to understand the cycle of violence, and its potential health impacts. Following this, the second session focuses on common health problems caused by IPV, mental health consequences, the purpose of managing stress, and techniques for managing stress. The third session emphasizes on strategies to help women manage their situation and develop their own customized problem management strategies. This session aims to improve participants’ ability or skills to solve and manage practical problems that may be influenced by other factors or changed by conflict in the family or unsolvable problems, such as employment. These problems are managed considering the participants’ existing personal strengths, resources, or support. This is followed by exploring safety-planning behaviors and preparing a customized safety plan in the fourth session. The last session involves exploring social support and assisting participants in planning to strengthen the social support identified. The contents of DeVI are detailed in Table 1.

**Table 1:** Contents of DeVI^a^.

Intervention session	Contents of PM+^b^ [16]	Additional components of DeVI
Session 1: understanding the muticomponent intervention	Introduction and confidentiality Before-intervention PSYCHLOPS^c^ assessment	General information about DeVI IPV^d^ and domestic violence, the cycle of violence, and its common impacts on health
Session 2: common consequences of IPV and domestic violence and managing stress	During-intervention PSYCHLOPS assessment and general review Purpose of managing stress Explanation and demonstration of some common stress management techniques Strategies to manage stress	Information about common health problems associated with IPV/domestic violence Details about the common mental health consequences of IPV/domestic violence Case of a women experiencing IPV/domestic violence
Session 3: managing problems	During-intervention PSYCHLOPS assessment and general review Strategy for managing problems and its usefulness Practice of the problem-managing strategy through a case example Application of the strategy in solving practical problems identified by participants Plan to solve the identified problems Practice of stress management techniques	Behavioral activation, during which participants will be encouraged to gradually re-engage with pleasant and task-oriented activities to improve mood and functionality
Session 4: development of a safety plan	During-intervention PSYCHLOPS assessment and general review	Safety behavior assessment Safety-planning behaviors applicable to the participants’ context Barriers to and facilitators of using safety behaviors Preparation of a safety plan in times of need Information about applying for legal protection orders Filing criminal charges
Session 5: strengthening social support and referrals	During-intervention PSYCHLOPS assessment and general review Available social support Assisting participant in making plans and approaches for strengthening social support identified	Distribution of an information booklet that details updated contact information or locally available organizations that offer different services to women experiencing IPV

^a^DeVI: Domestic Violence Intervention.

^b^PM+: Problem Management Plus.

^c^PSYCHLOPS: Psychological Outcome Profiles.

^d^IPV: intimate partner violence.

### Procedure

**Figure 3:**
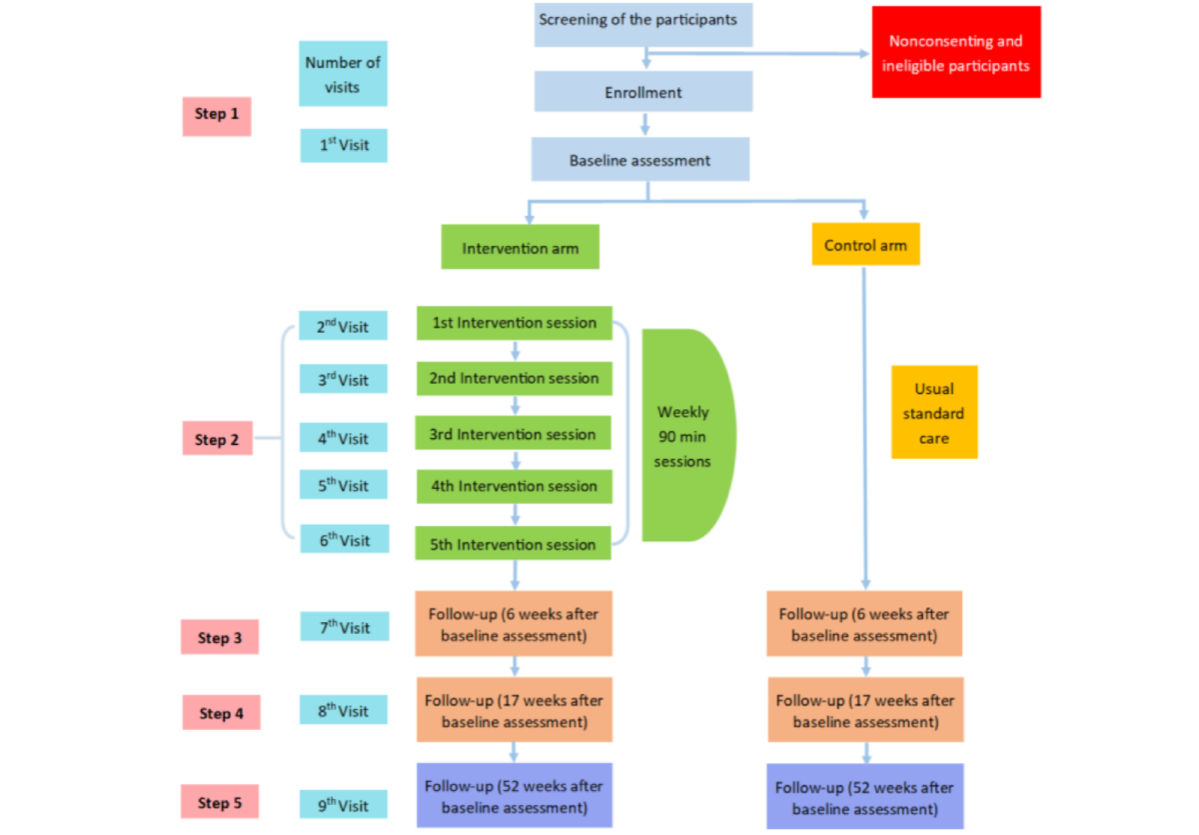
Flowchart of the study process.

Trained ANMs and nurses will be referred to as data collectors and intervention implementers, respectively. Their role throughout the study will be to approach women visiting health care facilities and screen them using a questionnaire for willingness to participate and eligibility. The study process is illustrated in Figure 3.

Data collectors will provide detailed information about the study to potential eligible participants and obtain written informed consent for a baseline assessment. Once consent is obtained, data collectors will conduct a baseline assessment. The baseline assessment will take about 30-45 minutes, which will be carried out in a separate room at the health care facilities. Participants will be made aware that refusal to participate will not have an impact on any type of support that they receive outside of the study. The detailed steps of study activities are listed in Table 2.

**Table 2:** Detailed steps of study activities at the cluster level.

Steps and arms		Timing	By whom	Determined by	Rationale	Outcome
**Screening**						
	Both intervention and control arms	N/A^a^	Data collectors	Data collectors	To screen eligible participants for the study. Data collectors will inform participants about the study and obtain written informed consent.	N/A
**Enrollment and baseline data collection**						
	Both intervention and control arms	N/A	Data collectors	Data collectors	Baseline data collection will help track the impact of the intervention.	Assessment of primary outcomes (psychological distress and occurrence of IPV^b^/domestic violence) at baseline
**DeVI^c^**						
	Intervention arm only	90 minutes/week for 5 weeks	Intervention implementers	Intervention implementers	To implement the intended intervention in the intervention arm.	N/A
	Intervention arm only	After session 5	Data collectors	Data collectors	To assess participants’ perception toward the intervention.	Exit interview to assess participants’ satisfaction and suggestions
**Follow-up**						
	Both intervention and control arms	6, 17, and 52 weeks after baseline in both arms	Data collectors	Data collectors	To compare baseline data with follow-up data between control and intervention arms.	Impact of the intervention on primary outcomes and secondary outcomes, such as a change in social support
**Follow-up of absconding participants**						
	Both intervention and control arm	N/A	Female community health volunteers	Data collectors and intervention implementers	Female community health volunteers to contact absconding participants over the phone or in person to reduce the attrition rate as much as possible.	Assessment of other outcomes, such as reasons for absconding

^a^N/A: not applicable.

^b^IPV: intimate partner violence.

^c^DeVI: Domestic Violence Intervention.

#### Intervention Arm

After the baseline assessment, study participants will be referred by data collectors to intervention implementers, whose role is to deliver the intervention package once a week, with each session lasting around 90 minutes. The intervention package aims to prevent the recurrence of IPV/domestic violence and address psychological distress among participants. During each session, intervention implementers will administer the Psychological Outcome Profiles (PSYCHLOPS) form, a mental health and social functioning tool. Before-intervention PSYCHLOPS data will be collected in session 1, and during-intervention PSYCHLOPS data will be collected in sessions 2-5. At the end of each session, the intervention implementer will schedule the participant’s next session. The intervention implementers will guide and encourage the participants to develop personalized plans and strategies for managing their problems, create a safety plan, and fully use existing community organizations and social support.

#### Enhanced Standard Care or the Control Arm

Control arm participants will receive standard care provided by the local health care facilities, depending on what they seek care for, and they will also receive an information booklet. Any participant with severe psychological distress or at risk for suicide will immediately be referred to a psychiatrist. Psychiatrists can provide remote counselling over the phone to the participant and, if necessary, also visit the participant in person. The study team will arrange the time, schedule, and consultation fees for the psychiatrist in both study arms, particularly for severe cases involving suicidal ideation identified during screening. Further, if the participant prefers to visit a psychiatrist herself, she will be provided with the travel cost.

#### Information Booklet

Both intervention and control arms will receive an information booklet that contains updated contact information for referral services in the study area. Further, they will receive information about the psychological distress resulting from IPV/ domestic violence and available services in the local context for psychological and legal support. Ahead of the study, the study team contacted and coordinated with concerned NGOs or community organizations working in the field of prevention of violence against women or management of mental health problems in the study area to ensure that women have safe places to visit in case of an emergency or difficulty.

#### Follow-up Assessments

Follow-up surveys will be conducted at 3 time points: 6 weeks after baseline assessment (ie, 1 week postintervention), 17 weeks after baseline assessment (ie, 12 weeks postintervention), and 52 weeks after baseline assessment (ie, 47 weeks postintervention). The data collection tools for follow-up will be similar to those for baseline assessments. However, in the last follow-up, participants will be asked questions and will be expected to contribute to the assessment of any sustained effects of DeVI on the prevention of revictimization and improvement in coping strategies against IPV. An exit interview will be conducted after intervention session 5.

IDIs will be conducted with health care providers from the intervention arm during or after the intervention to explore their experiences and perspectives of DeVI and program implementation. Health workers will be mobilized to identify suitable participants in the community, and those who provide written informed consent will be contacted for confidential interviews, preferably at the health care facility, both during and after the intervention.

### Adverse Events and Risk Management

There are some potential risks to participants, such as escalation of violence, as they reside at the same household as the perpetrator. Participants will be briefed about safety behaviors and contact numbers of local authorities and the study team (ie, health care providers, psychiatrists) that they may seek help from when in need. If any participants are in grave danger and do not want to go home, they will be referred to local shelter homes managed by different community organizations and NGOs.

If any other unprecedented harm or risk arises affecting the involved participants in any study arm, data collectors and intervention implementers from each site will notify the project coordinator immediately and manage the situation per guidance from the study team, depending upon the intensity of the adverse event. Adverse events would include a marked increase in suicidal thoughts, the death of the participants, suicide attempts, serious violence, the need for prolonged hospitalization, disability/incapacity, and any other life-threatening events. The study team will also assess these factors throughout the study period.

Any participant who is at risk for severe injury or who displays severe psychological distress and needs help from a specialist will be referred to a psychiatrist by the study team. Intervention implementers will receive the necessary help and guidance from the study team for risk management at respective sites. If any adverse event is reported by a participant or observed at any stage of the study by the data collector or intervention implementer, the study team will record all the necessary details in an adverse reporting form. This form, which is designed by integrating the adverse event form developed by WHO and the National Institutes of Health (NIH), will be used to capture adverse event details of individual participants throughout the study [25].

### Outcomes Measures

#### Primary Outcomes

The primary outcomes are psychological distress and the frequency and intensity of IPV/domestic violence. IPV/domestic violence will be assessed using the modified WHO multicountry questionnaire [36]. This instrument synthesizes women's experience of physical, sexual, and psychological violence, as well as controlling behavior by husbands/male partners, and also measures the severity of IPV/domestic violence. Psychological distress will be measured with the GHQ-12 [35] and the 9-item Patient Health Questionnaire (PHQ-9) [37]. Both tools have been translated and clinically validated in Nepal. The GHQ-12 has a cut-off score of 0.5, with sensitivity 85.6%, specificity 75.8%, positive predictive value 86.7%, and negative predictive value 84% [38]. Similarly, the PHQ-9 has a cut off score of ≥10, with sensitivity 94%, specificity 80%, positive predictive value 42%, and negative predictive value 99% [39].

#### Secondary Outcomes

The secondary outcomes are safety planning, use of community resources, social support, impaired functioning, symptoms of PTSD, personally identified problems, and health service use. Health care providers’ capacity to identify IPV/domestic violence to provide women experiencing IPV/domestic violence with risk mitigation services will also be assessed. Details of instruments and assessments for primary and secondary outcomes, including follow-up time, are provided in Tables 3-5.

**Table 3:** Overview of instruments and assessments for specific objectives concerning women experiencing IPV^a^/domestic violence.

Study objective and tool			Baseline (T_1_)^b^		Follow-up after baseline				
					6 weeks (T_2_)		17 weeks (T_3_)		52 weeks (T_4_)
**Objective 1:** **Assess the feasibility of DeVI^c^ in health care facilities in Nepal by exploring the views of key stakeholders, health care providers, and local women in the community.**									
	Formative assessment (details have been mentioned in the *Formative Assessment and Consultation Workshop* section)	0^d^		0		0		0	
**Objective 4: Measure the effectiveness of DeVI in addressing and resolving psychological distress, reducing the occurrence of IPV, and developing safety strategies for women experiencing IPV.**									
	Husband/male partner/domestic violence assessment	1^e^		1		1		1	
	GHQ-12^f^ [35]	1		1		1		0	
	Hospital Anxiety Depression Scale [40], PHQ-9^g^ [37], PTSD^h^ CheckList – Civilian Version, Multidimensional Scale of Perceived Social Support [41], WHO^i^ Disability Assessment Schedule [42]	1		1		1		0	
	Safety Behavior Checklist, use of community resources	1		1		1		1	

^a^IPV: intimate partner violence.

^b^T: time point.

^c^DeVI: Domestic Violence Intervention.

^d^0: not applicable.

^e^1: applicable.

^f^GHQ-12: 12-item General Health Questionnaire.

^g^PHQ-9: 9-item Patient Health Questionnaire.

^h^PTSD: posttraumatic stress disorder.

^i^WHO: World Health Organization.

**Table 4:** Overview of instruments and assessments for objective 5 (measure the impact of training health care providers on DeVI^a^ on their knowledge, attitudes, skills, and stigma toward IPV^b^ and the social and health-related consequences of IPV) concerning health care providers.

Tools	Pretraining	Posttraining	Follow-up 1 (T_2_)^c^	Follow-ups (T_3_ and T_4_)
Modified PREMIS^d^ [43]	1^e^	1	0^f^	0
MAKS^g^, Social Distance Scale [44], perceived dangerousness of the mental health patients [44]	1	1	0	1
In-depth interviews with health care providers	0	1	1	1

^a^DeVI: Domestic Violence Intervention.

^b^IPV: intimate partner violence.

^c^T: time point.

^d^PREMIS: Physician Readiness to Manage Intimate Partner Violence Survey.

^e^1: applicable.

^f^0: not applicable.

^g^MAKS: Mental Health Knowledge Schedule.

**Table 5:** Overview of instruments and assessments for specific objectives concerning health care providers and women experiencing IPV^a^/domestic violence.

Study objective	Tool	Session						Follow-up	
		1	2	3	4	5	1 (T_2_)^b^		2 (T_3_)
Objective 4: Measure the effectiveness of DeVI^c^ in addressing and resolving psychological distress, reducing the occurrence of IPV, and developing safety strategies for women experiencing IPV.	PSYCHLOPS^d^ [16]	1^e^	1	1	1	1	1		1
Objective 3: Explore participants’ and health care providers’ experiences with DeVI and the opportunities and challenges encountered during implementation.	Process evaluation	0^f^	0	0	0	0	1		1
Objective 3: Explore participants’ and health care providers’ experiences with DeVI and the opportunities and challenges encountered during implementation.	Exit interview of participants	0	0	0	0	1	0		0

^a^IPV: intimate partner violence.

^b^T: time point.

^c^DeVI: Domestic Violence Intervention.

^d^PSYCHLOPS: Psychological Outcome Profiles.

^e^1: applicable.

^f^0: not applicable.

#### Other Outcomes

Health care providers will be asked to respond to questionnaires, such as the modified Physician Readiness to Manage Intimate Partner Violence Survey (PREMIS), the Mental Health Knowledge Schedule (MAKS), the Social Distance Scale, and the perceived dangerousness of mental health patients pre- and posttraining to assess the impact of training on the health care providers’ knowledge and attitude. Further, MAKS and the perceived dangerousness of mental health patients will be assessed during integrated multicomponent supervision (approximately 90 days posttraining). Exit interviews will be conducted among 180 participants by data collectors in the absence of intervention implementers after the completion of all 5 sessions in a separate room by adopting a mixed methods approach. This will help determine changes postintervention and any aspects that need to be changed. This will also help assess the satisfaction and acceptability of DeVI from the participant’s point of view.

### Quantitative Data Collection for the Trial

Quantitative data will be collected electronically using REDCap software [45] and various data collection tools by data collectors in computer-based tablets at different time points. If any technical problem arises during electronic data collection, paper-based data collection will be performed. Each health care facility will be provided with a computer-based tablet with an updated questionnaire for data collection.

Data collection tools will include structured surveys and standard validated tools. The structured surveys will include questions on participants’ personal information (date of birth, ethnicity, address, contact information, and preferred time to call) and sociodemographic characteristics. The data collection tools in this study are standard international tools widely used and validated in different countries. However, some terminologies and contents have been contextualized to suit the Nepali context. The data will be collected at 4 different time points, once at baseline and at 3 follow-up time points (see Figure 3 for details). In addition, the PSYCHLOPS form will be filled in at the 5 intervention sessions.

### Qualitative Data Collection

Qualitative interviews will be conducted by purposively selecting participants to learn more about their perspectives toward DeVI. IDIs will be conducted with randomly selected intervention implementers to understand their challenges in implementing the intervention sessions and their suggestions for improving the applicability and sustainability of DeVI. A deeper understanding of their needs will help the study team alter the intervention approach and activities in the future. The qualitative process assessment will also help assess the acceptability of DeVI, which is key for scale-up and integration into routine services.

### Data Management and Analysis

#### Quantitative Data Analysis

Electronically collected data will be securely stored in a central office cloud, accessible only to designated study team members. Paper-based data pertaining to the study will be secured in a locker and retrieved from data collectors during central study team monitoring. Regular checks will be conducted to ensure data accuracy and quality, with necessary feedback provided to the data collectors. The primary measure of intervention effects will be based on intention-to-treat analyses. Multilevel analysis will be conducted to account for DeVI’s impact on primary and secondary outcomes, adjusting for clustering at the health center level [46]. The model will consider intervention × time interactions to assess the differential effects of DeVI on changes in outcomes from baseline to the final follow-up. Cluster-level variables will also be compared between intervention and control arms since randomization is based on health care facilities. Furthermore, the study will assess variations in outcome measures of interest between urban and rural areas.

The knowledge, perception, and preparedness of both intervention implementers and data collectors will be assessed before and after the training through pre-, post-, and follow-up tests. Quantitative data will be analyzed using STATA 15 software.

#### Qualitative Data Analysis

Qualitative interviews will be conducted in Nepali and audio-recorded with the participants’ consent. The recordings will be stored on a password-protected device. Transcription will be carried out in Nepali, and then the transcripts will be translated into English. The transcripts will undergo thorough review and validation against audio recordings to ensure accuracy. Qualitative content analysis will be performed. Independent researchers will initially conduct open coding, followed by discussion within the study team. Agreed-upon codes will be grouped into categories and themes, which will be developed and finalized based on the study objectives.

### Monitoring and Evaluation

The monitoring committee will comprise a study principal investigator, coinvestigators, and other independent experts who will oversee the study’s implementation and data collection. Any deviations from the protocol will be identified and documented during monitoring. The team will monitor and supervise field activities and communicate over the phone every day and visit all the study sites every month. The monitoring activities will be aided by checklists, tools, and manuals. Regular communication, both oral and written, will facilitate ongoing monitoring and updates among data collectors, intervention implementers, and other study members at the central offices.

### Process Evaluation

Concurrently with the trial, we will conduct process evaluation following the implementation component of the UK Medical Research Council’s guidance [47] in order to enhance comprehension of the DeVI’s acceptability among both women experiencing violence (reach) and health care providers. Additionally, we will assess the study’s adherence to the intended plan. The evaluation also aims to explore the barriers to and facilitators of DeVI (adaptations) from the perspective of health care providers, providing information about the possibility of its future sustainability and scalability, considering its effectiveness.

This process evaluation is based on the hypothesis that health care providers can identify women experiencing violence with mild-to-moderate psychological distress and that they can deliver planned counseling sessions and inviting women for further sessions and follow-ups on predetermined dates and times. This evaluation also hypothesizes that health care providers will allocate time for delivering the sessions in intervention sites, as well as conducting baseline and follow-up assessments in both intervention and control sites, in addition to their regular duty.

A mixed methods approach will be used for the process evaluation. Qualitative data will be collected through in-depth interviews with data collectors and intervention implementers at different time points. Quantitative data will be collected using an observation checklist and a 3-month follow-up period. The IDIs with health care providers will explore their experiences in, barriers to, and facilitators of recruiting and retaining women, as well as providing suggestions for improvement. Similarly, the observation checklist will help track their progress and status in the trial thus far, while the practice questionnaire will assess changes in their practice regarding violence identification and management. The practice questions will be derived from the practice section of the PREMIS questionnaire, which was previously used during training as a pretest. Qualitative data will be analyzed using thematic analysis [48], while quantitative data will be simply analyzed using descriptive analysis.

The process evaluation will uncover unforeseen insights, propose solutions, and introduce innovative approaches that encompass diverse aspects and perspectives of health care providers engaged in the trial. Moreover, should the trial deviate from its predetermined trajectory, we will diligently work to enhance the program by addressing areas requiring improvement, facilitating, overcoming barriers, ensuring resource availability, and effectively managing the time of health care providers.

Periodic monitoring will involve conducting site observations, observing intervention sessions with participant consent, and keeping track of participants. Regular meetings will be held between the study team and field teams to exchange updates and monitor progress. Adverse events related to the intervention will be reviewed periodically. The study team will cross-check the collected data for accuracy and completeness. Any identified errors during data monitoring will be promptly addressed through communication with the field teams for correction.

### Dissemination of Findings

Study findings, along with policy recommendations for enhanced collaboration between key stakeholders and increased coverage of IPV/domestic violence and mental health services for women, will be disseminated to relevant provincial and national stakeholders and authorities. The study results and recommendations will reach a wider audience through policy briefs, press releases, workshops, conferences, and scientific publications.

## Results

The trial received funding from the Swedish Research Council in 2019. The goal is to recruit 456 eligible women in each arm. As of December 29, 2022, a total of 269 eligible women were enrolled from the intervention sites and 309 eligible women from the control sites. The trial is currently in the recruitment phase. Data collection is expected to be completed by December 2023. Data analysis will begin shortly after obtaining all the data following the established plan. Regular data cleaning and monitoring are being conducted every day.

## Discussion

### Summary

This cluster-randomized trial aims to implement and evaluate the acceptability, feasibility, and effectiveness of DeVI in addressing psychological distress and enabling the secondary prevention of violence for women experiencing IPV. These interventions will be administered by trained nonspecialist mental health care providers in Madhesh Province, Nepal, at the health care facility level. As of December 29, 2022, the study had enrolled 59% (n=456) of eligible women from the intervention sites and 68% (n=456) of eligible women from the control sites. In low-income settings, such as Nepal, mental health specialists are scarce. Therefore, if this study proves effective, it could provide scientific evidence supporting the integration of mental health services into routine health care facilities in Nepal. This integration aims to prevent the recurrence of violence and address mental health issues among survivors of violence. Additionally, the study can offer valuable insights into how nonspecialist mental health care providers can address mental health problems and violence among women experiencing IPV/domestic violence. DeVI aims to improve access for marginalized women, particularly those who are hard to reach and have experienced IPV/domestic violence, while facing mental health challenges. Throughout the intervention, health care providers and survivors of violence will collaborate actively to address violence and enhance the women’s mental well-being. They will develop customized strategies for self-management of these issues. Investing in women’s health is considered a cost-effective strategy to improve the nation’s overall well-being [49].

We hypothesize that addressing the needs of women experiencing IPV/domestic violence will enhance their mental stability and reduce long-term health risks associated with violence. This study will contribute to bridging existing knowledge gaps by generating scientific evidence for regional policy makers, given the limited availability of data on mental health issues in resource-constrained settings.

The results from DeVI will hopefully support the integration of mental health services into routine health programs in Nepal. It could also add evidence to the global knowledge base on how and whether nonspecialist mental health care providers can be mobilized in addressing mental health problems in settings where IPV/domestic violence prevalence is high and human resources scarce. In addition, if this cluster-randomized trial is proven effective, this concept can be replicated in other LMICs following contextual adaptation and testing in each setting and target population.

### Limitations

Women experiencing violence and eligible for our study may refrain from providing consent due to the fear that their husbands or male partners will subject them to further abuse upon discovering their participation. The dropout rate may be higher due to the associated stigma and the fear of family members, society, and perpetrators. In a patriarchal society, such as Nepal, women are typically accompanied by their in-laws or husbands when seeking basic services at health care facilities, which creates reluctance to disclose issues related to violence or participate in the study.

### Conclusion

This trial aims to present vital evidence for mitigating the impact of intersecting violence and mental health issues among women in Nepal. If the intervention is found to be effective, the study has the potential to offer significant contributions and insights into how nonspecialist mental health care providers can address problems related to IPV and mental health among survivors in low-income settings.

## Abbreviations

ANM: auxiliary nurse midwife
DeVI: Domestic Violence Intervention
GHQ-12: 12-item General Health Questionnaire
FGD: focus group discussion
IDI: in-depth interview
IPV: intimate partner violence
KII: key informant interview
LMIC: low-and middle-income country
MAKS: Mental Health Knowledge Schedule
NGO: nongovernmental organization
PHQ-9: 9-item Patient Health Questionnaire
PM+: Problem Management Plus
PREMIS: Physician Readiness to Manage Intimate Partner Violence Survey
PSYCHLOPS: Psychological Outcome Profiles
PTSD: posttraumatic stress disorder
WHO: World Health Organization

## Data Availability

Data sharing is not applicable to this paper as no data sets will be generated or analyzed during this study.
